# Alcohol as poison: a narrative review of social science scholarship relevant to methanol poisoning in low- and middle-income countries

**DOI:** 10.1093/alcalc/agaf018

**Published:** 2025-04-28

**Authors:** Janet E Perkins, Knut Erik Hovda, Fazle Rabbi Chowdhury, Jane Brandt Sørensen, Michael Eddleston, Alice Street

**Affiliations:** Department of Social Anthropology, School of Social and Political Science, University of Edinburgh, Chrystal Macmillan Building, 15a George Square, Edinburgh EH8 9LD, United Kingdom; Dept of Acute Medicine, Oslo University Hospital, Kirkeveien 166 Bygg 3 0450, Oslo Norway; Institute for Clinical Medicine, University of Oslo, Søsterhjemmet, Kirkeveien 166, 0450 Oslo, Norway; National Poisons Information Centre, National Institute of Health, Lovisenberggata 8, 0456 Oslo, Norway; Department of Internal Medicine, Bangabandhu Sheikh Mujib Medical University (BSMMU), Shahbag, Dhaka-1000, Bangladesh; Global Health Section, Department of Public Health, University of Copenhagen, Øster Farimagsgade 5, 1353 Copenhagen K, Denmark; Centre for Pesticide Suicide Prevention, and Pharmacology, Toxicology and Therapeutics, Centre for Cardiovascular Science, Queen's Medical Research Institute, 47 Little France Crescent, University of Edinburgh, Edinburgh EH16 4TJ, United Kingdom; Department of Social Anthropology, School of Social and Political Science, University of Edinburgh, Chrystal Macmillan Building, 15a George Square, Edinburgh EH8 9LD, United Kingdom

**Keywords:** methanol, toxicity, alcohol, consumption, health systems, anthropology, social science

## Abstract

Aims: Methanol poisoning is a tragic and avoidable health emergency that threatens life and often leads to irreversible disability. It primarily occurs when people unwittingly consume beverages contaminated with the chemical compound under the guise of alcoholic spirits. Although reliable data on its burden are unavailable, methanol poisoning is thought to be increasing globally, particularly in low- and middle-income countries. Current scholarship related to methanol poisoning draws almost exclusively from clinical and epidemiological research traditions. In this article, and in the absence of anthropological scholarship examining methanol poisoning specifically, we provide a narrative review of anthropological and social science literature that bears on this growing phenomenon. Methods: We bring key areas of anthropological thought and inquiry that coalesce around the social phenomenon of methanol poisoning in conversation with the clinical and epidemiological scholarship. Results: We begin with a biographical account of methanol, an unlikely character which has become omnipresent in the material world. We then turn to a social scientific examination of alcohol consumption, to which methanol poisoning is tethered. We pay special attention to alcohol consumption in Muslim-majority settings, where alcohol is often proscribed, but methanol-related incidents are common. Subsequently, we examine the scholarship related to health systems and technologies, which come to bear on diagnostic and treatment encounters for those who have consumed toxic alcohol. Conclusion: We argue that anthropological perspectives are urgently needed to contribute to a fuller understanding of methanol poisoning and to design socially sensitive clinical and public health responses to address this avertable scourge.

## Introduction

‘RUET (Rajshahi University of Engineering and Technology) Student among 4 Dead After Drinking Toxic Alcohol in Kushtia.’ This headline briefly appeared atop the Prothom Alo newsfeed, a Bangladeshi national news outlet, on 24 April 2023, the day after Eid El-Fitr. The news report was succinct, no longer than 150 words, recounting the event in which four men died and five others fell ill after consuming toxic alcohol in Kushtia district, situated along the western border with India. The names of the deceased were published, amongst them a university student from RUET, a prestigious institute of higher education located in a nearby district. Beyond his status as a student, he was also identified as active in student politics at the university. Several other newspapers reporting the event were approximately the same length. They proffered few additional details, adding only the event’s confirmation by the police officer-in-charge and the resident medical officer of Kushtia District Hospital, where those who had ingested the toxic substance sought medical treatment.

Reports of death and injury resulting from the consumption of toxic alcohol punctuate news media in Bangladesh, as it does in other countries (see i.e. Motamedi 2022; Chandrasekaran 2024). Occasionally, these events achieve international new media attention, most often when the poisoning affects tourists from high-income countries visiting low- and middle-income countries (LMICs), as recently occurred in Laos (Drury and Walker 2024). Despite the tragedy of events involving tourists, such reporting obscures the fact that poisoning resulting from toxic alcohol is a common, neglected occurrence throughout many LMICs.

Whilst the chemical culprit is not consistently named in this reporting, toxicologists would attribute much of the blame for morbidity and mortality resulting from toxic alcohol, particularly when it occurs in clusters such as in Kushtia, to methanol. Although it can also occur through inhalation and skin absorption, methanol poisoning primarily occurs through the ingestion of beverages contaminated with the chemical compound under the guise of alcoholic spirits (Hovda et al. 2017). As methanol mimics to a certain extent the effects of ethanol, the active chemical compound in alcoholic beverages, it is used with the intent to add intensity to alcoholic drinks, fabricate alcoholic drinks where such drinks are difficult to source, or reduce their cost (Jones 2021; Tian et al. 2022).

Methanol is itself non-toxic. However, whilst both methanol and ethanol are easily metabolized in the body by the same enzyme in the liver, the end product of methanol is formic acid or formate, which accumulates and results in toxic effects in the form of cell death (Jacobsen and Mcmartin 1986; Hovda et al. 2017). Whilst alcohol consumption, particularly when it is excessive, can lead to compromised health in the short and long term, the human body generally manages ethanol unproblematically—and ethanol-containing drinks are the most widely used psychotropic substances worldwide (Dietler 2006; Hockings and Dunbar 2019b). In contrast, even small amounts of methanol operate as a poison when introduced into the body. A dose as low as 30 ml is considered lethal, and lower doses are capable of permanently impairing health, often as blindness (Jones 2021; Tian et al. 2022). Somewhat counterintuitively, ethanol can be protective for those who ingest methanol, as it blocks the metabolism of methanol and prevents formic acid production. In the clinical setting, ethanol is used as a first-line treatment for methanol poisoning (discussed further below).

The global burden that methanol poisoning inflicts on human life, health, and flourishing is poorly understood. There are no systematic reporting mechanisms for methanol poisoning, and the condition is rarely tested for; thus, epidemiologists struggle to provide reliable estimates related to its prevalence and consequences (Askarian et al. 2021). However, many in the clinical toxicology and public health communities consider methanol poisoning to be on the rise, with permanent impairment and mortality concentrated in LMICs (Zyoud et al. 2015; Nekoukar et al. 2021). Whilst effective treatments for methanol poisoning are well established, these work best when initiated early based on a confirmed diagnosis. Tragically, in LMICs, the sequelae of methanol poisoning are often compounded by the scarcity of access to diagnostics and treatment (Nekoukar et al. 2021).

These considerations lead us to interrogate how we can apprehend methanol poisoning and in what ways clinicians and the public health community can effectively respond to this growing health emergency. To date, methanol poisoning–related scholarship is almost entirely located in clinical and public health research. There is a glaring absence of robust social science scholarship, such as that drawing from anthropological approaches and methods, exploring this phenomenon. In this absence, we carried out a narrative review of anthropological and closely related scholarship, e.g. from historians, sociologists, and science and technology studies scholars, that can contribute to understanding methanol poisoning as a socially embedded phenomenon. Narrative reviews allow for flexibility in approaching a body of scholarship that provides new perspectives on a topic (Sukhera 2022). In this review, we bring clinical and public health research into conversation with these arenas of thought that coalesce around methanol poisoning.

In the first section of this paper, we draw from a social lives approach (Appadurai 1988; Whyte et al. 2002) to sketch a biography of methanol, an unlikely character which has become omnipresent in the material world. We then turn to anthropological examinations of alcohol consumption, to which methanol poisoning is tethered, with a particular attention to alcohol in Muslim-majority settings where methanol poisoning is common. Subsequently, we examine the scholarship related to diagnosis and treatment, which comes to bear on health service encounters for those who have consumed toxic alcohol. Through this review, we appeal for anthropological and related social science examinations to methanol poisoning. This, we argue, can contribute to a more expansive understanding of this phenomenon and to better public health and clinical responses to addressing this social and medical condition.

### Methanol: a biography

We begin our foray by examining methanol through a biographical lens. What is methanol? How did it become globally abundant? In what ways has it come to be socially meaningful? Anthropological and historical approaches can contribute to answering these questions. Anthropology has a strong tradition of attending to the social lives of things, recognizing the values, meanings, and uses of objects as never stable, but always under negotiation and transformation as they shift through time and social space (Kopytoff 1986; Appadurai 1988; Whyte et al. 2002). A social lives approach to chemical things, such as methanol, is peculiar, as it involves perceiving things that cannot be seen. Such things, Michelle Murphy argues, are materialized in historically contingent ways to become recognized as objects in this world. It is through the lay and expert traditions and practices that these historical contingencies entail, she suggests, that chemical exposures come to be ‘perceptible or imperceptible, existent or nonexistent’ (Murphy 2006, p. 7). Drawing from a social lives approach, in this section we consider how methanol becomes perceptible and attend to its multiplicity as it is negotiated and transformed across time and social spaces.

Also known as wood alcohol, methanol was first recognized as a perceptible, existent thing by the Egyptians, which they found to be a valuable addition to a mixture for embalming corpses. Millennia later, in 1661, Robert Boyle succeeded in synthetically reproducing it through the distillation of boxwood. Yet another century and a half later, French chemists Jean-Baptiste Dumas and Eugene Peligot would determine its elemental composition as distinct from ethyl alcohol (Sheldon 2017). Even after the chemical distinctions of methyl and ethyl alcohol gained widespread acceptance amongst chemists, their differences, particularly in relation to their effects on the human body, were assumed to be minor and they were considered equally intoxicating in the early 1900s. The toxicity in humans resulting from methanol exposure was generally attributed to impurities which trailed in the finished product when distilling wood. This conception shifted around the 1930s when the harmful effects of ingesting or inhaling methyl alcohol were increasingly recognized, and methanol became understood as a toxin in its own right (Stiles 1977).

Methods for synthetically producing methanol became more sophisticated and easier to replicate throughout the century. Producers moved away from the inefficient distillation of wood and towards catalytic processes (Sheldon 2017). A wide range of elements were tested for producing methanol, many successful, leading to a rapid expansion of production over the twentieth century. Daniel Sheldon describes three ‘golden ages’ in the production of methanol, largely driven by the energy industry in search of alternatives to fossil fuel–based sources of energy (Sheldon 2017). Methanol production has been equally important for the chemical industry, featuring as a common component of multiple chemical products, from windshield washer fluid and gas line antifreeze to carburettor cleaner, copy machine fluid, and perfumes (Ashurst et al. 2025). Through this array of uses, the compound has trekked the globe and is now omnipresent.

In recent years, there has been a galvanization in methanol enthusiasm and expansions in production, which now spans Europe, North America, and Asia, with China and India as major players. Methanol is highly valued in the energy and chemical sectors due to its manufacturing versatility and multiple and mutable uses. Just as early experimentation in methanol production saw the testing of multiple elements, today, feedstocks—the sources from which methanol might be produced—incorporate non-renewable sources, such as coal and natural gas, as well as agricultural residue, solar, municipal solid waste, and waste CO_2_ (The Methanol Institute 2024). Based on imaginaries of relying on renewable energy sources for production, some circles in the energy sector view methanol as the key to a carbon-neutral future (Sethi 2022).

Outside the human body, methanol is not particularly dangerous. However, inside the body, its value is rapidly transformed. Chemically, methanol differs from ethanol, the principal chemical found in alcoholic beverages, in only a minor reconfiguration of the chemical structure: the presence of one rather than two carbon atoms (Jones 2021). Whilst excessive alcohol consumption can lead to both acute and chronic health problems, the human body is generally adept at metabolizing ethanol. In contrast, the human body is not physiologically equipped to safely metabolize methanol at any dose, and even small amounts can permanently impair health and threaten life when ingested.

When consumed, methanol is quickly absorbed into the bloodstream, where it is converted into formaldehyde and then rapidly into formic acid/formate (Jacobsen and Mcmartin 1986; Ashurst et al. 2025). Formic acid/formate interferes with the optic nerve, leading to visual impairment, sometimes to the point of blindness. It also disrupts the body’s acid–base balance, sometimes leading to metabolic acidosis and respiratory failure, which can result in death (Jones 2021). In contrast to many other substances for which their role as remedy or poison is tempered based on the dose (Arnold 2016, pp. 8–9), methanol is not used for any therapeutic purpose.

This travel point in methanol’s journey brings us full circle to our case study, which opened this article. Rarely do discussions of methanol’s promises and effects as a chemical substance outside the human body coincide with discussions of how it becomes visible when identified as the culprit of acute poisoning events when ingested inside the human body. Across chemical and energy sector scholarship, news coverage, and industry narratives, methanol is represented not only as harmless, but as a chemical inscribed with the aspiration of combatting some of the greatest threats facing humanity. In clinical and public health narratives, in contrast, methanol is an unmitigated poison, with exposure holding the potential to destroy the body. This multiplicity matters. It matters not only for anthropologists but also for policy and public health responses. How a thing and its effects become perceptible sets the limits for what is imperceptible and, thus, the parameters for what is desirable and possible.

### Alcohol, health, and society

Methanol poisoning occurs predominantly when individuals consume presumed ethylated beverages contaminated with methanol. Thus, methanol poisoning is best understood as embedded in the consumption of mind-altering substances. Public health narratives tend to approach alcohol consumption from a problem perspective, locating drinking problems as stemming from individual behaviours (Bennett and Cook 1996; Marshall et al. 2001). Whilst much of the public health research throughout the decades has tried to disentangle ‘normal’ drinking from ‘problem’ drinking and thereby design policy and intervention responses based on these categories (Bennett and Cook 1996), an abstinence approach has recently gained traction. This is reflected in the 2018 *Lancet* publication reporting that no level of alcohol consumption improves health (Burton and Sheron 2018), which has become the official position of WHO (WHO 2023). Such an approach risks collapsing unintentional consumption of methanol into a catchall category in which all alcohol consumption is considered harmful (see e.g. Shokoohi et al. 2020).

In contrast, anthropological scholarship contributes to expanding such narrow understandings of the roles of alcohol in society. As an alternative to public health approaches, a rich body of anthropological scholarship has approached alcohol production, distribution, and consumption as an inherently social and exceptionally widespread phenomenon (Heath 2012; Hockings and Dunbar 2019b). Indeed, archaeological evidence suggests that since the human body evolved to metabolize ethanol, no society with the capacity to produce ethanol-containing beverages has opted not to (Hockings and Dunbar 2019a). Alcohol production and consumption, even when proscribed, has always been socially and culturally valorized (Mandelbaum 1965; Dietler 2006; Heath 2012). As David Mandelbaum put it in 1965, ‘There have been very few, if any, societies whose people knew the use of alcohol, yet paid little attention to it. Alcohol may be tabooed; it is not ignored’ (Mandelbaum 1965).

As one of many forms of consumption of food, beverages, and other psychotropic substances, anthropologists recognize alcohol consumption as both reflecting and constituting social worlds (Dietler 2006, Douglas 2013 [1987], Heath 2012). Whilst alcohol, in its ubiquity, was often encountered and engaged with peripherally in early anthropological scholarship, it was during the 1970s and 1980s that an anthropology of alcohol was consolidated (Heath 1987). Throughout the 1980s, much of this work was carried out by medical anthropologists in the USA, and often as an appendage to epidemiological and public health studies on alcohol. Some of this anthropological work was applied, whilst others challenged popular strands of thought related to alcohol, such as the ‘disease model’ of alcohol abuse (Bennett and Cook 1996). In the 2000s, anthropological interest in alcohol continued unabated but moved beyond functionalist interpretations and analyses of how alcohol reflects society and culture, to considerations of alcohol *as* material culture and how social contexts are constituted through the production, distribution, and consumption of alcohol (Dietler 2006; Gamburd 2010; Hockings and Dunbar 2019b).

However, this scholarship has been reticent to examine alcohol in predominantly Muslim societies. This is a particular blind spot for understanding methanol poisoning, as outbreaks have been widespread across Muslim-majority countries (Shafi et al. 2016; Amin et al. 2017; Delirrad and Mohammadi 2020). Existing anthropological scholarship on alcohol in such contexts has tended to approach alcohol consumption as an aberration and peripheral to society (see i.e. Heath 2012). Such perspectives fail to account for the rich traditions of alcohol production and consumption across the Muslim world. Indeed, the English word ‘alcohol’ traces its etymology to the Arabic word *al-Kuhl*, and the innovation of the distillation of alcohol is credited to the Persians (Matthee 2023). Moreover, rather than strict abstention, scholars have noted Muslims historically demonstrating varying interpretations of the injunction against alcohol, and note that within some Islamic schools of thought, wine consumption is celebrated (Arab 2022; Matthee 2023).

Recent anthropological scholarship has called for a deeper engagement with alcohol in Muslim contexts, to consider how the presence of alcohol in such societies might be considered socially meaningful rather than an aberration (Arab 2022). Some social research has started to shed light on the social meanings of alcohol consumption across Muslim societies, e.g. in ‘postponing piety’ (Debrevec 2012), enacting resistance (Silverstein 2012), or in creating an ‘ideal world’ (Arab 2022, see also Douglas 2013 [1987]).

For example, Pooyan Tamimi Arab (2022) examines alcohol production, distribution, and consumption amongst Muslim migrants in the Netherlands as normalized within Muslim social spaces. His sensitive and nuanced account demonstrates how alcohol amongst these migrants served a dual purpose, at once producing an ‘alternate reality’ as they navigated a society often experienced as hostile and providing a framework in which they could negotiate identity along political, ethnic, and religious grounds. Liza Debevec explored the consumption of alcohol as amongst the practices engaged in by Muslim Burkinabés living in urban centres that seem to oppose their religious convictions. She demonstrates, however, that alcohol consumption allowed the Burkinbés to ‘delay piety’ until they reached an age and social status in life considered suitable for full observance of Islam. This act of postponement, she argues, ‘comes along with a clearly pronounced respect for the purity of Islam’ (Debrevec 2012, p. 44). Further interrogations along these lines can serve to understand how methanol features in alcohol social practices in these settings where alcohol is generally thought to be socially, ethically, and, in some cases, legally proscribed, yet likely serves important social purposes.

Even quieter than explorations of alcohol in Muslim-majority settings have been anthropologically informed explorations of toxic alcohol or methanol-contaminated alcohol in any setting. Indeed, despite its relative frequency, this particularly lethal dimension of alcohol in society has remained virtually untouched. One exception to this is found in Michelle Gamburd’s (2010) rich and nuanced examination of alcohol production, consumption, and distribution in Sri Lanka. Not being the focus of her work, however, the role of toxic alcohol is recognized but mentioned only briefly in a single section of the ethnographic monograph.

Research employing, drawing from, and expanding anthropological perspectives on alcohol production, distribution, and consumption present promising pathways for deepening the understanding of the conditions surrounding methanol poisoning. Such a lens can serve to unsettle the predominant global health narratives of all alcohol use as harmful and tend to the various ways alcohol constitutes identity and sociality in situated contexts. Through this, methanol poisoning can thus be better understood as a dangerous extension of socialities constituted by and through alcohol.

### Navigating methanol poisoning diagnosis and treatment

When presumed ethanol consumption slides into methanol ingestion, this brings us into new territory that often involves health responses and medical treatment. From a clinical perspective, even after exposure, most mortality resulting from methanol poisoning can be averted and much of the related disability—notably brain and eye damage (Sobhi et al. 2024)—minimized through well-established biomedical approaches. These are most likely to be successful when methanol poisoning is identified soon after exposure and treatment is initiated swiftly (Mcmartin et al. 2016; Hovda et al. 2017). However, diagnostic confirmation typically requires extensive laboratory testing, and given that its symptoms mirror those of other common health disorders—including shortness of breath, abdominal pain, nausea, weakness, and headache (Hovda et al. 2017)—diagnostic testing for methanol poisoning is generally considered only when an outbreak of methanol poisoning is suspected (Hovda et al. 2005). Tragically, by the time laboratory-based diagnosis is achieved, it is often too late for effective management in patients, resulting in death or permanent brain or eye damage (Jones 2021).

The bulk of scholarship related to the diagnosis and treatment of methanol poisoning takes the form of clinical case studies, examining individual cases or small clusters of poisoning (Barnard et al. 2014; Rostrup et al. 2016; Amin et al. 2017; Eskandrani et al. 2022; Krebs and Czarnecki 2022). These case study reports illuminate pathways of diagnosis and treatment from the time of admission in health facilities and focus on the clinical presentation of patients. They suggest that the clinical diagnosis of methanol poisoning is extremely complex, even when it unfolds in well-resourced health systems. In under-resourced health systems often characteristic of LMICs, diagnostic challenges are exacerbated, as the modern laboratory diagnosis is often slow or unavailable. In these case studies, diagnostic processes in LMICs often appear as failing to adhere to best practices, either due to a lack of materials and equipment or health service provider knowledge (Barnard et al. 2014; Rostrup et al. 2016).

Whilst there is no anthropological literature that examines the diagnosis and treatment of methanol poisoning specifically, anthropologists and other social scientists have developed a rich tradition exploring diagnosis as the achievement of practical and embodied processes (Mol 2002; Street 2014; Street and Kelly 2021). This scholarship demonstrates diagnostic processes as messy and sinuous, and as generating multiplicity and uncertainty as much as singularity and certainty. This lens can provide a path to step back from seeing diagnostic pathways to methanol poisoning as aberrant and lacking and provide sensitive and nuanced accounts of the people and technologies involved in generating diagnosis as an epistemological basis for guiding treatment. Such understandings are crucial for making sense of methanol poisoning diagnoses in LMICs, where diagnosis is generally achieved through history taking, clinical assessment, and diagnostic tests that detect conditions associated with methanol poisoning, such as metabolic acidosis. In health facilities, multiple health service providers and departments are often mobilized in these processes, expanding the potential for multiplicity in interpretations.

Furthermore, methanol poisoning has recently emerged in global health as an object amenable to the promises of point-of-care tests (POCTs)—diagnostic tools that do not rely on laboratories and promise rapid results (Pai et al. 2012; Drain et al. 2014). Some consider POCTs promising devices to shortcut the time to treatment initiation and guide health service provider decision-making for methanol poisoning in under-resourced settings. One such test has been designed to test formate concentration in the blood, and thus the presence of methanol, by using a sample of one drop of blood, and promising results within 5 min. This test is currently undergoing randomized controlled trials in India and Bangladesh to test for its sensitivity and specificity, as well as the feasibility of its integration into health systems (Hovda et al. 2021). However, as critical social science scholarship has shown us, diagnosis is never simple. It will be important to stay socially attuned to how the design and application of new diagnostic tools for detecting methanol poisoning reshape and are reshaped by the social and material conditions of diagnosis and care in situated settings.

Even with a clinical diagnosis, treatment pathways for methanol poisoning are not routinized in many settings, particularly in those where such poisoning most commonly occurs. The drug fomepizole, which blocks the metabolism of methanol to its toxic metabolite of formic acid, is recommended as the first-line treatment for methanol poisoning treatment (Skolnik and Wilcox 2013). Fomepizole is recognized for its effectiveness in treatment and its ease of use (Casavant 2001), and was added to the WHO Essential Medicines List in 2013 (WHO 2013). Where fomepizole is unavailable, ethanol may also be administered orally or intravenously, functioning physiologically the same way as fomepizole. However, treatment using ethanol is considered more complex, as it requires careful monitoring of the dose levels and patient responses, which can demand hourly withdrawal and testing of blood samples (Ashurst et al. 2025). Moreover, treatment with ethanol may also map onto other social and cultural dimensions of alcohol in society, described in the previous section. In contrast, fomepizole is generally administered using a standard dose every 12 h (Brent et al. 2001), or every 8 h or 4 h during dialysis (Lao et al. 2022).

Both options for treatment are far from simple in LMIC contexts. In many LMICs, health systems remain under-resourced and bear the legacies of colonialism and decades of vertical health programming (Street 2014). This often means that certain health issues receive a great deal of resourcing and attention whilst others remain marginalized. Methanol poisoning belongs to the latter: it has been marginalized in global health and national health programming. This means that effective treatment pathways, which also hinge on robust supply chains and health systems, are elusive. Anthropological scholarship has demonstrated the challenges of maintaining supply chains even for prioritized health issues such as malaria (see e.g. Umlauf and Park 2018), much less for health issues considered rare and niche. Whilst fomepizole is widely available throughout the USA and Europe, it remains scarce across LMICs (Mcmartin et al. 2017). Furthermore, even ethanol can be difficult to access in spaces where alcohol consumption is socially and legally proscribed, and understaffed health facilities may struggle to provide the careful monitoring required when treating methanol poisoning with ethanol.

Diagnosis and treatment of methanol poisoning may be further shaped by its layering with the social valuations of alcohol consumption, particularly in Muslim-majority settings. As it is entwined with alcohol consumption, methanol poisoning carries a moral weight with it that other health conditions, such as malaria, do not. In this sense, clinical encounters for those experiencing methanol poisoning may share similarities with other conditions that carry a negative moral valence, such as substance use disorders (Neale 1999; Ezell et al. 2021) or self-harm (Saunders et al. 2012). In this literature, scholars have demonstrated that stigma and negative attitudes of health service providers towards patients presenting with these health conditions compromise interpersonal interactions and medical care. With the negative moral valuation of alcohol consumption in settings where methanol poisoning occurs, this may come to bear on health service encounters.

This may be particularly true in Muslim-majority settings, where treatment for alcohol-related disorders may face additional hurdles. For example, Al-Ansari et al. (2020) carried out a case study of alcohol disorder treatment systems in Iran. In this setting, national political will to design and improve treatment for alcohol use disorders resulted in the design of robust policies and a strategy for this purpose modelled on international guidelines. However, the authors concluded that an inhospitable implementation environment, at least in part shaped by the social stigma surrounding alcohol consumption, meant that these policies and strategies remained largely nominal and unimplemented. To the best of our knowledge, no anthropological studies have been carried out to date specifically examining methanol poisoning diagnosis and treatment. However, this existing scholarship points towards ways to open up understanding of the diagnosis and treatment pathways of methanol poisoning in a trajectory of health and social experiences. Such attention can contribute to informing sensitive responses to addressing methanol poisoning in LMICs.

### A way forward

In the absence of anthropological and related scholarly explorations taking methanol poisoning as its object of inquiry, this paper provided a narrative review of arenas of social science thought that coalesce in the realm of the phenomenon. Current scholarly understandings of methanol poisoning are almost entirely composed of studies focusing on the clinical management of those presenting with the condition (Hovda et al. 2005; Nekoukar et al. 2021; Alhusain et al. 2024) and public health literature trying to trace this evasive epidemiological object (Askarian et al. 2021). Through this review, we have highlighted fields of anthropological and related enquiry which stand to bear on understandings of methanol poisoning.

In terms of its social lives, methanol as a substance is not only perceptible when it enters the human body; it has become a ubiquitous feature of what is commonly referred to in social science circles as the Anthropocene—described as the epoch in which we now live characterized by a fundamental human reshaping of the natural environment (Steffen et al. 2011). This reshaping has encompassed an amplification in the presence, embodied, and globalized flows of toxins (Cielemęcka and Åsberg 2019). Methanol can be seen as amongst the toxins that have prospered in the age of the Anthropocene, a chemical structure once confined to the minuscule space of boxwood which, through human action, became an easily synthesized compound that can be made from nearly anything and used for almost anything in the chemical and energy sectors. Based on its omnipresence, methanol can be thought of as part of what anthropologist Alex Nading refers to as ‘living in a toxic world’ (Nading 2020). Future research would benefit from understanding methanol as having social lives as it moves across time and space.

And yet, amongst the toxins that now make up our world, methanol is a relatively benign one—i.e. when it is safely confined outside the barriers of the human body where it usually stays. It is typically only unwittingly that the compound is taken into the body when someone has introduced methanol into a product intended for consumption. By stepping away from the teetotaling tendencies in public health discourses, anthropological research can contribute to understanding the nebulous space between alcohol as a normalized psychotropic substance and alcohol as poison—i.e. methylated spirits masquerading as uncontaminated ethyl alcohol. Human encounters with the latter bring us into new territories and often into formal health systems. Social science scholarship on methanol poisoning can at once contribute to a richer understanding of the sociality of care in biomedical health settings, the processes involved in achieving diagnosis and treatment, and people’s experiences with care. Such knowledge is urgently needed to improve clinical responses to methanol poisoning events in these settings.

We appeal for increased research drawing from anthropological approaches and perspectives that takes up methanol poisoning as its object of inquiry. In particular, we call for original research across LMICs in which methanol poisoning is prevalent and particularly in Musim-majority settings. We would encourage such work to consider methanol poisoning as one dimension of alcohol-related practices embedded within Muslim-majority societies. The research agenda we lay out here is not exhaustive, yet we hoped to draw attention to potential contributions of anthropology to better understanding and responding to methanol poisoning. Our research team is currently undertaking such a study embedded within a clinical trial to improve methanol poisoning diagnosis in Bangladesh. We urge for similar research across different settings.

## Conclusion

The toxic alcohol event we presented at the outset of the article was an avoidable tragedy. Epidemiological data have yet to generate reliable data on the toll methanol poisoning takes on human life and health, but numerous news reports, particularly in LMICs, suggest that the incidents that come to light are the tip of the iceberg, and that methanol poisoning is a growing scourge. In this paper, we have reviewed anthropological arenas of enquiry that can contribute to a fuller understanding of this urgent issue. Thus, our call is to expand on this rich scholarship and move forward on a research agenda that does not separate methanol poisoning from the social context in which it is embedded. To make sense of the methanol poisoning event in Kushtia and others like it, we need to approach it as a social and cultural problem, as well as a health problem. Such considerations will be crucial for designing public health interventions, tools, and policies for averting similar tragedies.

## Data Availability

Data can be made available by request to the corresponding author.
